# Does venture capital Quan Zi affect stock mispricing: Evidence from Chinese listed companies

**DOI:** 10.1371/journal.pone.0281255

**Published:** 2023-04-13

**Authors:** Ping Chen, Li Deng

**Affiliations:** 1 Institute of Chinese Financial Research Studies of SWUFE, Southwestern University of Finance and Economics, Chengdu, China; 2 School of Finance, Sichuan Vocational College of Finance and Economics, Chengdu, China; University of Durham: Durham University, UNITED KINGDOM

## Abstract

Chinese traditional culture is characterized by "Quan Zi" culture with a "differential order pattern". As a special informal institutional arrangement, "Quan Zi" plays an important role in the capital market. This paper investigates how Venture Capital Quan Zi affects the stock mispricing of invested companies. Using the syndicate investment data of China’s venture capital institutions from 2009 to 2019, this study documents that Venture Capital Quan Zi significantly increases the positive deviations of stock prices of Quan Zi-backed firms but has no obvious influence on the negative deviations, showing an asymmetric effect on stock mispricing. In addition, this effect is dynamic. Stock mispricing significantly increased in the lock-up period and the following year, but then gradually weakened. Mechanism tests suggest that, on the one hand, Venture Capital Quan Zi increases a company’s earnings manipulation, thus raising investors’ expectations to push up stock prices. On the other hand, Venture Capital Quan Zi boosts the stock price through market reaction channels, increasing institutional investors’ shareholdings, positive media coverage and stock liquidity. This paper has high theoretical and applied value to guide the orderly competition of capital and the supervision of institutional investors.

## 1. Introduction

Connections play an irreplaceable and crucial role in Chinese society. In recent years, the academic field has provided rich evidence for relationship research from the perspectives of political relations, hometown relations, and alumni relations. However, the research has ignored a key element: China’s interpersonal relationships are not simple "relationship networks" but a "Quan Zi" culture with a "differential order pattern" [1]. Deeply influenced by Confucian culture, Chinese interpersonal relationships are different from the Western "group structure" and form a "differential order pattern". In this kind of social structure, everyone forms a network with themselves at the center. The inner and the outer of Quan Zi represent different relationships of intimacy and trust, and the rules they follow are also quite different. Resources, technology, and information are shared only by coterie members.

In a highly uncertain industrial environment, venture capital (VC) syndications work together to reduce investment risk [2]. In the process of multiple syndicate investments, their interests are deeply tied, and Quan Zi is much easier to form [3, 4]. Existing studies equate "relationship" with "Quan Zi", confusing the concept of "Quan Zi" with "relationship", which cannot accurately reflect the connotation of "relationship" and the characteristics of social structure in Chinese society. In fact, "relationship" is only the surface, and "Quan Zi" plays a key role [5, 6]. There is an implicit assumption that the more complex the network, the richer the social resources. However, the reality is that the richer the network, the more likely it is to participate in "Quan Zi", and the top layers of Quan Zi have real influence. Granovetter [5] defined the four dimensions of the relationship from strong to weak connections, and believed that the higher the frequency of interaction, the deeper the emotional involvement, the stronger the intimacy and the higher the reciprocity. China is a society characterized by the "return of favors", trust and obligation. The "strong relationship" theory has revised the "weak relationship" hypothesis, and the advantage of "structure hole" has also been challenged [6]. Information and resources are embedded within the top layers of Quan Zi, which contributes to organizational cohesion and emotional support. The scarcity of resources determines that the "Quan Zi" has top-to-down layers, and the distinction of being inside and outside. The general " interpersonal relationship " and "Quan Zi" cannot be compared in terms of resource allocation, operation ability and market influence.

The phenomenon of "Quan Zi" in the capital market has gradually attracted the attention of many scholars [3, 4, 7, 8], but there are still many problems with "how to accurately measure Quan Zi culture". Existing research has not given satisfactory answers to the basic questions about the role and related mechanisms of "Quan Zi" in a transition economy. Relevant large-sample empirical studies are even scarcer.

To obtain investment returns, VC institutions need to gradually reduce their holdings in the secondary market. Therefore, how to successfully withdraw from investment projects has always been the focus of VC institutions. Since the income of VC institutions depends significantly on the performance commission, institutional managers have a strong incentive to pursue high returns under this salary structure and pay close attention to stock performance. At present, academia focuses on the price discovery function of VC institutions [9, 10], but often ignores the fairness problem derived from the capital Quan Zi to grab profits. Will VC Quan Zi push up stock prices through the power of capital, especially stock prices that seriously deviate from fundamentals? This is the starting point of our research.

In this case, the VC field was selected, which is prominent in the phenomenon of the capital Quan Zi as a natural experimental place. Using the syndicate investment data of Chinese VC institutions from 2009 to 2019, the proxy capital Quan Zi was constructed, which reflects the circle-stratification characteristics of capital syndicates. We further identified the information of Initial Public Offering (IPO) enterprises invested by VC Quan Zi in the year of listing and two years prior to listing. We also identified VC Quan Zi of each invested enterprise. Based on the perspective of stock mispricing, the impact of VC Quan Zi on the stock pricing of invested enterprises was studied.

Asset mispricing refers to the stock price deviating from its intrinsic value under various factors [11]. The literature generally agrees that two factors affect asset mispricing. First, based on the theory of information asymmetry, which finds the reasons within the company, we studied the quality of information disclosure [12, 13], earnings management [14] and other factors. Second, external drivers, such as investor cognitive bias [15, 16], limited arbitrage [17], short selling restrictions [18], media sentiment [19], etc. In fact, whether internal or external, the power of capital may not only promote the market value of enterprises to return to the basic value through channels but also overestimate the market value of enterprises. If VC Quan Zi corrects the price normally and rationally, it is undoubtedly conducive to improving resource allocation efficiency. However, in reality, ’’correction’’ is often excessive. Suppose VC Quan Zi pushes up stock prices through unreasonable channels, which induces the unfairness of capital misallocation. In that case, it will not only infringe on the benefit of ordinary investors and hinder the standardized development of emerging markets, but also affect the long-term stability of financial markets once the bad atmosphere of VC Quan Zi is formed, which is an urgent and important topic.

In this paper, evidence that VC Quan Zi has an asymmetric effect on the stock mispricing of Quan Zi-backed firms was documented. The stronger VC Quan Zi is, the more obvious the positive deviation. However, it has no significant effect on negative deviation. Compared with companies without VC Quan Zi, companies invested in VC Quan Zi significantly increased the positive deviation of asset mispricing by 2.2%. For each 1% increase in the number of VC Quan Zi, the company’s share price is overvalued by 3.1%. In addition, we measured the ability of VC Quan Zi to mobilize resources from three dimensions: investment quantity, investment amount and IPO quantity. The results show that the greater the strength of VC Quan Zi is, the more obvious the positive deviation of asset mispricing, which further confirms that VC Quan Zi pushes up stock prices. In addition, it was found that the impact of VC Quan Zi on asset mispricing has a dynamic effect over time. During the lock-up period and the following year, the stock price is significantly pushed up, and then the impact is weakened. This shows that VC Quan Zi has obvious opportunistic motivation. To minimize the endogenous effects, we used difference-in-difference (DID), propensity score matching analysis (PSM), and random sampling of the placebo test for estimation. The results are consistent, indicating that the study is robust.

VC Quan Zi can impact invested companies’ stock mispricing through a few plausible channels. VC Quan Zi not only participates in the company’s operation and management, but also uses market forces to affect stock prices. Therefore, the channels affecting stock mispricing from internal and external dimensions were examined. We found that VC Quan Zi pushes up stock prices through the internal channels of earnings management in the exit stage. In addition, VC Quan Zi boosts stock prices through three external factors: positive media exposure, increased institutional investors holding, and increased stock liquidity. Finally, in the robustness test part, we replaced the measurement method of asset mispricing and add other control variables. We also referred to Tian [2], Yang et al. [20], and Yin et al. [21] and measured the excess abnormal returns of company stock prices from the perspective of market anomalies using CAPM (1964), the Fama-French three-factor model (1993), and the Carhart four-factor model (1997) for the robustness test. We further confirmed the mispricing effect of VC Quan Zi based on its benefits, exacerbating the deviation of enterprise market value from its intrinsic value.

The innovations and contributions of this paper are mainly reflected in the following two aspects. First, the study expands the research framework of the VC syndication field. It is a valuable supplement to the literature on how VC plays a role in the capital market after the company goes public and influences its market value addition. Prior studies have paid more attention to the role of VC firms in the private stage of a company. At present, the literature on the impact of VC participation on a company’s capital market performance using real transaction data is still scarce. On the one hand, VC in China has not attracted enough attention from scholars because of the lack of data. On the other hand, VC as a private fund is limited to internal communication, management, and other information. At present, the conclusions of empirical studies on the above issues are quite different [10, 22–24], and there are relatively few studies focusing on the perspective of VC syndications [25, 26]. In addition, there is little literature exploring the role of VC Quan Zi on the market value of the invested company from the perspective of stock asset mispricing. Given the background of the Chinese "Quan Zi" culture with a "differential order pattern", this paper investigates how VC Quan Zi affects the stock mispricing of invested companies and explores how VC Quan Zi can drive up the market value of enterprises under the drive of profit. We attempted to uncover the mechanism of VC Quan Zi’s participation in capital market pricing and to trigger thinking about efficiency and fairness.

Second, this study enriches the literature on the factors that affect asset mispricing. Prior studies have paid more attention to internal and external factors. The external factors are mainly from the perspectives of noise traders, limited arbitrage, short-selling restrictions, and behavioral finance [15, 17–19]. The internal reasons are mainly from the perspectives of the management’s earnings activities, the quality of accounting information disclosure, and the mandatory disclosure of CSR reports [12, 14, 27, 28]. There is little literature focusing on VC as a special shareholder behavior. The particularity of VC lies in that it can not only participate in the operation and management of the company, but also influence the external market through multiple channels. Quan Zi formed by VC syndication is likely to have different effects on the pricing of the capital market. Does it remove the impure from the pure or add fuel to the flame? This paper suggests that VC Quan Zi is an important factor that affects stock mispricing and provides important theoretical and practical guidance for promoting the healthy development of the VC industry and capital market.

The remainder of this paper is organized as follows. Section 2 proposes the hypothesis. Section 3 presents the data and variable construction and reports the summary statistics. Section 4 to Section 6 report the main empirical results and examine plausible underlying economic channels and a battery of robustness checks. Section 7 provides the conclusion and policy implications.

## 2. Literature review and research hypothesis

As a "financial anomaly", asset mispricing challenges efficient market theory. Under the theory of the efficient market hypothesis (EMH), information transfer is timely and accurate, investors are perfectly rational, all valuable information is fully reflected in stock prices, and the price can accurately reflect the stock’s intrinsic value. In perfect efficient markets, there are two understandings of asset mispricing. One believes that asset mispricing is a temporary phenomenon. The price will soon return to its intrinsic value due to the existence of arbitragers [29]. The other is that asset mispricing is regarded as part of compensation for the systematic risk that cannot be explained by asset pricing models [30]. However, the mispricing phenomenon of a stock price deviating from its intrinsic value is common in mature and emerging capital markets. As China’s capital market is emerging and transitional, the phenomenon of stock overvaluation is more obvious [31]. Research that examines the causes of asset mispricing mainly attributes the causes to two categories. One focuses on the external market and carries out research on the aspects of noise traders, limited arbitrage, short-selling restrictions, and behavioral finance [15, 16, 18, 19, 32]. Another considers both external market and internal corporate factors. Based on asymmetric information theory, these pieces of literature analyze the impact of management’s earnings management activities and accounting information disclosure on asset mispricing [27, 28, 33, 34].

Against the background of Chinese culture with a "differential order pattern", an increasing number of collaborative syndicate factions are emerging [35]. VC Quan Zi is formed based on real investment relationships with multiple syndicate investments. After repeated screening and approving, they form more trusting and loyal patterns. Although VC institutions have the efficiency function of price discovery [10, 36], VC Quan Zi’s pursuit of interests will probably lead to fairness problems. At different stages, VC Quan Zi is likely to show different interest demands and behavior rules. For example, their "self-benefit" motivation and "win-win" economic benefit supplement each other before they successfully pass the listing verification. Scholars have recognized that VC institutions have the value-added effect of "self-benefit" and "altruism" coexisting in the process of "investment and management". For example, they enhance the value of invested enterprises through professional management experience, extensive resource networks, participation in strategy formulation, operating management, and corporate governance [37, 38]. Moreover, they grasp the best market opportunity and help the invested enterprises to a timing listing [39]. However, these are the basis for a smooth exit. Only by successfully exiting during the fund contract period, obtaining high returns, and distributing benefits to limited partners (LPs) in a timely manner can VC institutions establish or maintain their reputation and raise funds steadily. After a successful listing, VC institutions will try to create favorable conditions for an exit [40]. They may have conflicts of interest with invested companies due to their inconsistent goals. Therefore, VC Quan Zi will primarily pursue the value-added effect of capital’s "self-benefit" motive.

On the one hand, VC Quan Zi has deeply bound interests, high goal consistency, and a strong voice in the enterprise. There are the same value concepts and behavioral norms within the "Quan Zi". Consistent standards and attitudes can stimulate members’ cooperative tendencies to maintain shared values. Furthermore, it can also help to establish expected goals [41]. This goal promotes trust in relationships and structural stability in Quan Zi. Members of Quan Zi are assigned specific roles, rights, and obligations. They use the standards and rules within Quan Zi to further the same value identification and loyalty and then protect the interests of small groups. If VC Quan Zi forms certain powers, they may threaten or conspire with management to drive up stock prices [42]. found that company managers have the motivation to push up or maintain high stock prices and engage in earnings manipulation for selfish purposes, such as building their empires and obtaining higher remuneration [40]. found that companies invested by VC institutions have higher earnings management than companies without VC support after the listing. The main purpose of VC institutions is to create favorable conditions for a smooth exit.

On the other hand, VC Quan Zi has more market power under the social influence of various members. The interpersonal relationship of "Quan Zi" is investment-oriented and profitable [43]. Due to the investment in long-term trustworthy relationships, the relationship of "Quan Zi" members is closer and more loyal. Thus, it is more convenient and effective to mobilize the resources of individuals or groups in the relationship. The signal display function of "Quan Zi" can attract the attention and pursuit of more highly qualified investors and market participants, such as media and institutional investors, and render optimistic market sentiment.

China’s capital market started late, and it is still in an emerging developmental and transitional period. On the one hand, the participation threshold is relatively high, the trading variety is limited, and the short-selling mechanism has not been established maturely, so most investors can only rely on rising prices to make profits. On the other hand, the opacity of information in China’s capital market is still high. Medium and small investors have immature investment experience and limited information acquisition channels. They often rely on public opinions and market rumors and lack subjective rational judgment. It is easy for them to imitate highly qualified investors’ behavior and follow the "dominant opinionated climate", buying high and selling low. Therefore, the most favorable situation is expected for VC Quan Zi to successfully reduce its holdings and exit after the listing to exacerbate the upward deviation of stock prices by mobilizing Quan Zi’s resources as much as possible. Trying to play Quan Zi partners’ important role of "mutual help" renders and signals the favorable value information of the company, thus promoting the overvaluation of the company stock prices. At the same time, it will trigger the optimism of market investors and raise resonance, leading to a further rise in stock prices driven by the herd effect. Conversely, in theory, when a company’s stock price is undervalued, VC Quan Zi may have an incentive to return the stock price to its intrinsic value, reducing the degree of asset mispricing. However, the reality is that when stocks are undervalued for a variety of reasons, investors are likely to react more emotionally, leading to further price declines. Kahneman and Tversky [44] found that investors prefer to take risks when faced with losses. As a result, VC Quan Zi’s cost is likely to be disproportionate to the return. Alternatively, there will be little effect on reducing holdings and exiting. It is expected that when the market is undervalued, VC Quan Zi is likely to choose to give up the intervention. Therefore, the following hypothesis is proposed.

**Hypothesis 1A.** Keeping everything else unchanged, VC Quan Zi will push up the company’s stock prices and aggravate the positive deviation of asset mispricing. However, it has no significant effect on the negative deviation of asset mispricing.

VC Quan Zi can exert the service and supervision functions well; thus, the reciprocal relationship and effort degree among members are improved under the role of "Quan Zi" [45–47]. Meanwhile, Quan Zi can better provide value-added services and help to improve the operational efficiency of the invested company. The cooperation of familiar partners in Quan Zi can avoid "free riding" and innovation-grabbing problems to a certain extent. If VC Quan Zi continues to play the "certification" role in the IPO exit stage, it will strengthen the supervision of the company and reduce earnings management [48, 49]; restrain overinvestment and alleviate underinvestment [50]; improve the governance structure of the board of directors and the level of corporate governance [51]; and reduce agency costs and the degree of information asymmetry, which is conducive for external investors to explore companies’ intrinsic value. When the company’s value is overvalued or undervalued, VC Quan Zi will play a role in correcting the deviation, reducing asset mispricing, and helping the company’s market value return to its intrinsic value. Therefore, the following hypothesis is proposed.

**Hypothesis 1B.** Keeping everything else unchanged, VC Quan Zi will make the company’s stock price return to its intrinsic value when the price is overvalued or undervalued, reducing the degree of asset mispricing.

## 3. Research design

### 3.1. Data

This paper uses data on China’s VC institutions from 2009 to 2019. The data come from the three sub-libraries of "Institution", "Investment Event" and "Exit Event" in PEdata. According to Wu et al. [50], there is no obvious distinction between venture capital (VC) and private equity (PE) in China. Therefore, VC institutions whose institutional types are both PE and VC were selected from the PEdata "institutions" sub-library for research. In line with common practice, we excluded "undisclosed venture capital firms", "Investors with undisclosed information", "unidentified investees" and "undisclosed invested capital" in the "Investment Event" sub-library. We mainly explored the influence of VC Quan Zi on stock mispricing in China’s A-share capital market. Hence, we further identified the information of IPO enterprises supported by VC Quan Zi in the year of listing and two years prior to listing from "Investment Events" and "Exit events" sub-libraries. VC Quan Zi is constructed using the method described in Section 3.2. Other information, such as the company’s stock price performance and financial data, was obtained from the CSMAR (China Stock Market & Accounting Research Database). In this paper, all continuous variables are winsorized at the top and bottom 1% to eliminate the influence of extreme values. Finally, the information of VC Quan Zi and IPO companies was matched. Following the literature [19, 33], we used quarterly data to calculate asset mispricing indicators. In the process, we excluded the samples with obvious errors in accounting information, negative book value and no disclosure of financial information, and excluded the samples with trading suspended due to asset reorganization, merger and acquisition. Our final sample includes 7739 observations representing 829 firms.

### 3.2. Variable definition and descriptive statistics

#### 3.2.1 Variable definition

*3*.*2*.*1*.*1 Venture Capital Quan Zi of listed companies*. This paper investigated VC institutions’ direct strong connections formed by self-centered and multiple co-investment behaviors. According to the syndicate investment history information of VC institutions and following Hochberg et al. [52], this paper defines that in a certain period of time, venture capital institution B, which has co-invested with venture capital institution A for many times, is A’s Quan Zi, and constructs the proxy of venture capital Quan Zi. When constructing VC Quan Zi proxy, we refer to Hochberg et al. [52], identifying VC institutions’ pairwise syndicate investment relationships by rolling the time window. Whereas our major innovation is that we fully consider the practical application of China. First, Hochberg et al. [52] adopted a 5-year window period. However, we adopt a 3-year window period instead, considering that the duration of VC funds in China is shorter than that in the United States. The length of the window period allows enough time to determine the partners with specific preferences and avoids containing old information. Second, the trust structure of Chinese society is different from that of western society. The universal orientation of "general trust" is replaced by the specific orientation of "special trust". Its characteristic is that the boundary of the trusted scope is a closed network, focusing on interpersonal trust within Quan Zi. The VC Quan Zi proxy we construct is based on repeated syndicate investment relationships of VC institutions, rather than occasional and loose syndicate investment relationships, reflecting "differential order pattern" of Quan Zi’s inside and outside.

We use the following methods to construct VC Quan Zi and identify the capital "Quan Zi" of listed companies.

First, using the information on syndicate investments in the past three years, we identify partner institutions B, C, and D that have invested with VC institution A three times or more. Based on this, we identify A-B, A-C, and A-D as A’s "Quan Zi", respectively.

Second, we calculate the number of A’s self-centered "Quan Zi" (A-B, A-C and A-D) to measure the breadth of institution A’s "Quan Zi". Then, we calculate the sum of the investment quantity, investment amount, and IPO quantity of A’s Quan Zi partners (B, C, and D) in the past three years to measure the power of institution A’s "Quan Zi". Then, we adopt the rolling window method and reidentify each window period to obtain the dynamically adjusted VC Quan Zi proxy.

Third, we determine the information of IPO companies supported by VC institutions in the sample period. We match the information of companies with VC Quan Zi in the IPO year and two years before the IPO.

In practice, several core elements are used to investigate and evaluate the competitiveness and comprehensive performance of VC institutions, including investment ability, investment results, exit returns and other dimensions. In the empirical analysis, investment amount, investment quantity, and successful IPO quantity can be used as important indicators to measure the strength and power of VC institutions [2, 53–55]. To comprehensively analyze VC Quan Zi of listed companies, we construct three types of variables. The first is the dummy variable of whether the company is invested in by VC Quan Zi (*StkQzDum*). If the company has more than or equal to one VC institution that is supported by Quan Zi, it is equal to 1; otherwise, it is 0. The second is the number of "Quan Zi" of the company (*StkQzNum*). The third is the power of "Quan Zi". Following the literature [2, 53–55], the power of Quan Zi is measured by the logarithmic investment quantity (*StkInvNum*), logarithmic investment amount (*StkInvAmt*) and logarithmic IPO quantity (*StkIpoNum*) of the sum of the Quan Zi partners of venture capital institution A in a certain period.

*3*.*2*.*1*.*2 Measurement of asset mispricing*. Because it is difficult to observe a company’s intrinsic value directly, how to measure asset mispricing is a difficult problem for empirical finance. We adopt the residual income valuation method established by Feltham and Ohlson [56] for measurement. This method estimates the intrinsic value of the company according to the company’s core financial indicators, and the noise is small when estimating. Therefore, it is widely recognized and adopted by academia [13, 19, 57]. In the robustness test, following the calculation method of [58], we use the average industry level to calculate asset mispricing. Following Tian [2], Yang et al. [20], and Yin et al. [21], we measure the excess abnormal return of company stock prices from the perspective of market anomalies. CAPM (1964), the Fama-Frech Three-factor Model (1993) and the Carhart Four-factor Model (1997) are used for verification.

The first method is calculated as follows. In the first step, we calculate residual income according to Eq (1).

$$RR_{it}=EPS_{it}-r_{t}*ROS_{it}$$ (1)

where *RR*_*it*_ is the residual income of company i in period t, *EPS*_*it*_ is the earnings per share of common stock of company i in period t, *r*_*t*_ is the discount rate, with the one-year risk-free rate of return as a proxy variable, and *ROS*_*it*_ is the net asset value per share of company i in period t.

The second step is to regress Eqs (2) and (3) and extract coefficients *α*_1_, *α*_2_, and *β*_1_.


$$RR_{i,t+1}=\alpha_{1}+\alpha_{2}RR_{it}+\alpha_{3}ROS_{it}+\varepsilon_{i,1t+1}$$ (2)

$$ROS_{i,t+1}=\beta_{1}ROS_{it}+\varepsilon_{i,2t+1}$$ (3)

The third step is to calculate the parameter values of *γ*_1_, *γ*_2_, and *γ*_3_ ccording to Eq (4).

where

$$\gamma_{1}=\alpha_{1}/\left[\left(1+\text{r}-\alpha_{2}\right)*r\right],\gamma_{2}=\alpha_{2}/\left[\left(1+\text{r}-\alpha_{2}\right)*r\right],\gamma_{3}=\alpha_{2}/\left[\left(1+\text{r}-\beta_{1}\right)*r\right]$$ (4)

The fourth step is to substitute *γ*_1_, *γ*_2_,*γ*_3_ into Eq (5) to calculate the intrinsic value of the company *V*_*it*_.


$$V_{it}=\gamma_{1}+\gamma_{2}RR_{it}+\gamma_{3}ROS_{it}$$ (5)

Finally, we divide the stock price *P*_*it*_ and the intrinsic value of company *V*_*it*_ in the same period and take the natural logarithm. Then, we obtain the degree of asset mispricing of company i in period t (Mis1).


$$\text{Mis}1=\text{ln}\left(\frac{P_{it}}{V_{it}}\right)$$

If the degree of asset mispricing (Mis1) is positive, it indicates that the stock price deviates upward from the intrinsic value of the company; if it is negative, it indicates a downward deviation from the intrinsic value of the company. The greater the absolute value of the degree of asset mispricing (Mis1), the higher the degree of deviation.

*3*.*2*.*1*.*3 Control variables*. Following the literature [2, 14, 28, 33], the following widely used variables were adopted as control variables: the natural logarithm of total assets at the end of the quarter (*Size*), return on equity of the company at the end of the quarter (*ROE*), the quarterly growth rate of the company’s main business income (*Growth*), market to book ratio (*BM*), shareholders’ equity concentration at the end of the quarter (*Hhi*), the logarithm of the number of board members (*BoaNum*), and the dummy variable of whether the company belongs to the high-risk industry (*HigInd*), the logarithm of the number of years from the company’s establishment to IPO (*Age*), the natural logarithm of China’s economic policy uncertainty index (*Epu*), the leverage ratio of the company at the end of the quarter (*Lev*), the proportion of the company’s independent directors (*IndBNum*), the shareholding ratio of the controlling shareholder (*First*), etc. At the same time, in the robustness test, following You and Wu [19], we added the closing price at the end of the last quarter (*CloP*), the Investor Sentiment Index (*Mood*), and the quarterly negative deviation index calculated by the daily rate of return (*Ncskew*) as control variables. Thus, we further excluded some possible omitted factors that will influence the degree of stock mispricing, such as the stickiness of stock prices (momentum or reversal), irrational behaviors of investors triggered by sentiment in the market, and unstable stock prices.

#### 3.2.2 Descriptive statistics

Table 1 shows the descriptive statistics of the main variables. As expected, the influence of VC Quan Zi on positive and negative asset mispricing is likely to be asymmetric. In the empirical part, the influence of VC Quan Zi on positive and negative asset mispricing was studied respectively.

**Table 1:** Descriptive statistics. This table reports descriptive statistics for all dependent and independent variables used in the paper.

Panel A: *Mis1>* = 0					
Variable	Mean	Min	Max	SD	Obs
*Mis1*	0.638	0.000	3.110	0.471	5481
*StkQzDum*	0.732	0.000	1.000	0.443	5481
*LSQzNum*	0.746	0.000	2.773	0.559	5481
*LSInvNum*	5.791	0.000	10.870	3.619	5481
*LSInvAmt*	9.022	0.000	16.110	5.577	5481
*LSIpoNum*	3.409	0.000	8.006	2.290	5481
*Age*	2.686	1.609	4.127	0.333	5481
*Size*	21.370	19.560	25.650	0.847	5481
*Lev*	1.608	1.011	28.710	0.842	5481
*Growth*	0.192	-2.064	42.400	1.013	5481
*ROE*	0.056	-0.406	0.742	0.059	5481
*BM*	0.230	0.013	1.164	0.120	5481
*First*	0.34	0.075	0.811	0.138	5481
*BoaNum*	2.129	0.000	2.996	0.331	5481
*IndBNum*	0.369	0.000	0.750	0.115	5481
*Hhi*	0.179	0.026	0.661	0.103	5481
*Epu*	5.907	4.303	6.765	0.694	5481
*HigInd*	0.292	0.000	1.000	0.455	5481
Panel B: *Mis1<*0					
Variable	Mean	Min	Max	SD	Obs
*Mis1*	-0.293	-1.410	0.000	0.211	2258
*StkQzDum*	0.683	0.000	1.000	0.466	2258
*LSQzNum*	0.696	0.000	2.079	0.552	2258
*LSInvNum*	5.420	0.000	10.210	3.798	2258
*LSInvAmt*	8.383	0.000	15.010	5.829	2258
*LSIpoNum*	3.319	0.000	7.083	2.446	2258
*Age*	2.722	1.609	3.714	0.339	2258
*Size*	21.360	19.520	25.010	0.751	2258
*Lev*	1.734	1.014	14.020	0.908	2258
*Growth*	0.818	-0.991	12.940	2.727	2258
*ROE*	0.050	-0.692	0.318	0.051	2258
*BM*	0.503	0.154	1.904	0.194	2258
*First*	0.341	0.073	0.777	0.126	2258
*BoaNum*	2.110	0.000	3.045	0.389	2258
*IndBNum*	0.364	0.000	0.750	0.126	2258
*Hhi*	0.168	0.025	0.609	0.089	2258
*Epu*	6.004	4.303	6.765	0.785	2258
*HigInd*	0.109	0.000	1.000	0.311	2258

Panel A of Table 1 represents the descriptive statistics of positive deviation of asset mispricing degree (*Mis1*> = 0). The average degree of asset mispricing (*Mis1*) calculated by the residual income valuation method is 0.638. The maximum degree of asset mispricing (*Mis1*) is 3.11, indicating that some companies’ market prices have been greatly inflated. In terms of the explanatory variables, among IPO companies co-invested by VC institutions, an average of 73.2% of the sample companies have VC Quan Zi’s support. This indicates that the "Quan Zi" phenomenon is very obvious in the VC industry. The mean value of the logarithm of Quan Zi’s number is 0.746. "Quan Zi" partners’ power shows great differences. On average, the logarithm of "Quan Zi" partners’ investment quantity is 5.791, the logarithm of investment amount is 9.022, and the logarithm of IPO quantity is 3.409.

Panel B of Table 1 represents the descriptive statistics of negative deviation of asset mispricing degree (*Mis1*<0). The sample size is 2258, which is much smaller than that of the positive deviation of asset mispricing (5481). This is consistent with the reality of China’s capital market. The phenomenon of stock price overvaluation is more prominent in our sample companies. The average degree of asset mispricing (*Mis1*) is -0.293. In terms of the explanatory variables, an average of 68.3% of the sample companies have VC Quan Zi’s support. The mean value of the logarithm of Quan Zi’s number is 0.696. The mean value of "Quan Zi" partners’ power for the logarithm of investment quantity, the logarithm of investment amount and the logarithm of IPO quantity is 5.42, 8.383, and 3.319, respectively.

## 4. Main findings

### 4. 1 Venture capital Quan Zi and asset mispricing

To verify our hypothesis, we run the regression on Eq (6), where Mis1_it_ is the company’s stock mispricing at the end of the quarter, VC Quan Zi (*X*_*it*_) that we focus on is characterized by three types of variables. That is the dummy variable of whether the company is invested by VC Quan Zi (*StkQzDum*), the logarithm of the number of Quan Zi (*StkQzNum*), and the power of Quan Zi. To measure “power”, we use the logarithmic investment quantity (*StkInvNum*), the logarithmic investment amount (*StkInvAmt*), and the logarithmic IPO quantity (*StkIpoNum*) as proxy variables. The higher the value of the proxy variable is, the greater the power of “Quan Zi”.

Following Pantzalis and Park [33], Wang and Zhu [14], and Xu [28], we select the following widely used variables as control variables: the natural logarithm of total assets (*Size*), return on equity (*ROE*), market to book ratio (*BM*), shareholders’ equity concentration (*Hhi*), the logarithm of the number of board members (*BoaNum*), the logarithm of the number of years from the company’s establishment to IPO (*Age*), the leverage ratio of the company (*Lev*), the proportion of the company’s independent directors (*IndBNum*), and the shareholding ratio of the controlling shareholder (*First*). In addition, VC institutions are keen to invest in risky industries. However, high risk and economic policy uncertainty will affect every aspect of the market, thus affecting asset mispricing. Therefore, the dummy variable of whether the company belongs to a high-risk industry (*HigInd*) and the logarithm of China’s Economic Policy Uncertainty Index (*Epu*) are added as control variables in this paper. We also control for industry and year fixed effects. Tables 2 and 4 report the regression results of VC Quan Zi on the positive and negative asset mispricing samples, respectively.


$${Mis1}_{it}=\beta_{0}+\beta_{1}X_{it}+{\beta_{2}Control}_{it}+{Industry}_{i}{\text{+Year}}_{t}+\varepsilon_{it}$$ (6)

**Table 2:** Venture Capital Quan Zi and asset mispricing (positive).

	(1)	(2)	(3)	(4)	(5)
*VARIABLES*	*Mis1*	*Mis1*	*Mis1*	*Mis1*	*Mis1*
*StkQzDum*	0.022***				
	(2.780)				
*LSQzNum*		0.031***			
		(4.481)			
*LSInvNum*			0.003***		
			(3.591)		
*LSInvAmt*				0.002***	
				(3.571)	
*LSIpoNum*					0.004***
					(2.709)
*Age*	-0.027**	-0.024**	-0.027**	-0.027**	-0.026**
	(-2.426)	(-2.230)	(-2.432)	(-2.445)	(-2.383)
*Size*	0.344***	0.340***	0.344***	0.344***	0.344***
	(32.431)	(31.550)	(32.336)	(32.345)	(32.370)
*Lev*	-0.137***	-0.136***	-0.137***	-0.137***	-0.137***
	(-5.491)	(-5.485)	(-5.498)	(-5.495)	(-5.493)
*Growth*	-0.012***	-0.012***	-0.012***	-0.012***	-0.012***
	(-2.629)	(-2.611)	(-2.617)	(-2.616)	(-2.617)
*ROE*	-1.788***	-1.775***	-1.789***	-1.790***	-1.787***
	(-16.635)	(-16.580)	(-16.665)	(-16.661)	(-16.662)
*BM*	-3.464***	-3.455***	-3.463***	-3.463***	-3.462***
	(-43.834)	(-43.784)	(-43.864)	(-43.867)	(-43.800)
*First*	-0.274***	-0.289***	-0.275***	-0.274***	-0.276***
	(-3.296)	(-3.490)	(-3.308)	(-3.306)	(-3.321)
*BoaNum*	0.020*	0.019*	0.020*	0.020*	0.019*
	(1.782)	(1.686)	(1.801)	(1.817)	(1.746)
*IndBNum*	-0.051	-0.047	-0.050	-0.050	-0.048
	(-1.527)	(-1.410)	(-1.496)	(-1.518)	(-1.435)
*Hhi*	0.459***	0.489***	0.463***	0.462***	0.463***
	(3.919)	(4.178)	(3.959)	(3.953)	(3.952)
*Epu*	-0.051***	-0.051***	-0.051***	-0.051***	-0.051***
	(-4.106)	(-4.145)	(-4.107)	(-4.109)	(-4.110)
*HigInd*	0.055***	0.055***	0.055***	0.055***	0.056***
	(5.673)	(5.683)	(5.615)	(5.611)	(5.728)
*Constant*	-5.453***	-5.359***	-5.438***	-5.440***	-5.445***
	(-25.854)	(-25.005)	(-25.765)	(-25.775)	(-25.794)
*Year*	YES	YES	YES	YES	YES
*Industry*	YES	YES	YES	YES	YES
*Observations*	5,481	5,481	5,481	5,481	5,481
*R2*	0.699	0.700	0.699	0.699	0.699

This table reports the OLS regression results on the relation between VC Quan Zi (*StkQzDum*, *LSQzNum*, *LSInvNum*, *LSInvAmt*, *LSIpoNum*) and stock mispricing (*Mis1*) for the samples with the positive deviation of asset mispricing (Mis1> = 0). All variables are defined in Table 3. Year and industry effects are controlled for in all columns. Robust t statistics are reported in the parentheses below the coefficient estimates, with ***, **, and * denoting significance at 1%, 5%, and 10%, respectively.

**Table 3:** Variable definitions.

	Variables	Variable Definition	Calculation Method
Dependent Variables	*Mis1*	The degree of asset mispricing	The residual income valuation method by Feltham and Ohlson [56]
Independent Variables	*StkQzDum*	The dummy variable of whether the company is invested by venture capital Quan Zi	If yes, it equals 1. Otherwise, it equals 0
	*LSQzNum*	The logarithm of the number of venture capital Quan Zi	
	*LSInvNum*	The logarithm of the number of companies invested by venture capital Quan Zi	
	*LSInvAmt*	The logarithm of the amount invested by venture capital Quan Zi	
	*LSIpoNum*	The logarithm of the number of successful IPOs by venture capital Quan Zi	
	*Size*	The natural logarithm of total assets	
	*ROE*	Return on equity	Quarterly net profit divided by quarterly net assets
Control Variables	*Hhi*	Shareholders’ equity concentration	
	*BoaNum*	The logarithm of the number of board members	
	*HigInd*	The dummy variable of whether the company belongs to a high-risk industry	If yes, it equals 1, Otherwise, it equals 0
	*First*	Shareholding ratio of the controlling shareholder	
	*Age*	The logarithm of the number of years from the company’s establishment to IPO	
	*Epu*	The natural logarithm of China’s economic policy uncertainty index	
	*BM*	Market to book ratio	Quarterly market value divided by quarterly book value
	*Lev*	Leverage ratio of the company (%)	Quarterly total debt divided by quarterly total assets
	*IndBNum*	Proportion of the company’s independent directors	
	*Growth*	Quarterly growth rate of the company’s main business income	

Table 2 reports the regression results. The coefficients of VC Quan Zi (*X*_*it*_) are positive and statistically significant, indicating that VC Quan Zi improves the positive deviation of stock mispricing. As shown in Column (1), compared with companies without Quan Zi, the degree of stock mispricing of companies with VC Quan Zi (*StkQzDum*) significantly increases by 2.2%. Column (2) shows that a 1% increase in the number of Quan Zi (*StkQzNum*) increases asset mispricing by 3.1%. Columns (3) to (5) show that the power of capital “Quan Zi” also significantly boosts stock prices. When the investment quantity (*StkInvNum*), the investment amount (*StkInvAmt*), and the IPO quantity (*StkIpoNum*) increased by 1%, the degree of stock mispricing increased by 0.3, 0.2, and 0.4 percentage points, respectively.

The results in Table 2 support Hypothesis 1A. VC Quan Zi pushes up the market valuation of the company and makes the market value of the company deviate from its basic value to a greater extent, exacerbating the positive deviation of asset mispricing. In terms of the control variables, the coefficients of company Size (*Size*) are significantly positive in Columns (1) to (5), indicating that the larger the size of the company, the more asset pricing deviates from the basic value. The coefficients of the dummy variable of whether the company belongs to the high-risk industry (*HigInd*) are significantly positive, suggesting that the degree of asset mispricing of companies in high-risk industries is higher. However, the coefficients of China’s Economic Policy Uncertainty Index (*Epu*) are all significantly negative, indicating that when economic policy uncertainty is low, stock price overvaluation is more serious.

As shown in Table 4, the coefficients of VC Quan Zi in Columns (1) to (5) are generally statistically insignificant. The results indicate that VC Quan Zi has no statistically significant impact on the negative deviation of asset mispricing. This may be due to the high cost of raising the price and the small effect of selling out when stock is undervalued. First, a large proportion of Chinese stock investors are retail investors, who tend to chase popular stocks and discard unpopular ones [59]. Therefore, once the stock price is undervalued after listing, even if VC Quan Zi utilizes a lot of resources to raise the price, it is difficult to change the preconceived mentality of investors. Second, the best option for VC Quan Zi is to pool resources on raising the price of overvalued stocks in this case, which is also conducive to protecting their interests when they exit. Even if the undervalued stock returns to its intrinsic price, VC Quan Zi’s return will not be as good as pushing up the overvalued stock’s return. In a cost-benefit trade-off, VC Quan Zi chooses to raise the price of overvalued stocks and abandon undervalued ones. Third, it is difficult for undervalued shares to return to their intrinsic value after an IPO in China. From an investment point of view, though, buying these shares at a low price will yield higher returns when their value returns in the future. However, Chinese investors are more retail investors, and their investment style tends to be short-term and conceptual [60], Investors are unable to effectively identify the stocks with potential investment value, which leads to the undervaluation of the stock prices for a long time [61]. Therefore, VC Quan Zi’s influence on the undervalued stock price is not significant. This asymmetric effect is consistent with our expectations of Hypothesis 1A. In the following part of this paper, we focus on how VC Quan Zi affects the positive deviation of asset mispricing.

**Table 4:** Venture capital Quan Zi and asset mispricing (negative).

	(1)	(2)	(3)	(4)	(5)
*VARIABLES*	*Mis1*	*Mis1*	*Mis1*	*Mis1*	*Mis1*
*StkQzDum*	0.005				
	(0.860)				
*LSQzNum*		-0.006			
		(-1.189)			
*LSInvNum*			0.001		
			(0.878)		
*LSInvAmt*				0.001	
				(0.940)	
*LSIpoNum*					0.001
					(0.725)
*Age*	0.011	0.010	0.011	0.011	0.011
	(1.187)	(1.167)	(1.208)	(1.201)	(1.208)
*Size*	0.223***	0.224***	0.223***	0.223***	0.224***
	(19.992)	(19.986)	(20.005)	(19.988)	(20.026)
*Lev*	-0.125***	-0.125***	-0.125***	-0.125***	-0.125***
	(-10.253)	(-10.208)	(-10.258)	(-10.256)	(-10.257)
*Growth*	0.001***	0.001***	0.001***	0.001***	0.001***
	(6.550)	(6.982)	(6.721)	(6.668)	(6.760)
*ROE*	-0.739***	-0.745***	-0.738***	-0.738***	-0.7386***
	(-4.426)	(-4.473)	(-4.421)	(-4.422)	(-4.423)
*BM*	-1.054***	-1.056***	-1.054***	-1.054***	-1.055***
	(-23.351)	(-23.272)	(-23.366)	(-23.356)	(-23.391)
*First*	-0.168**	-0.167**	-0.169**	-0.168**	-0.170**
	(-2.108)	(-2.089)	(-2.121)	(-2.115)	(-2.134)
*BoaNum*	0.001	0.001	0.001	0.001	0.001
	(0.172)	(0.146)	(0.175)	(0.177)	(0.177)
*IndBNum*	0.018	0.017	0.018	0.018	0.018
	(0.737)	(0.701)	(0.729)	(0.727)	(0.730)
*Hhi*	0.404***	0.396***	0.405***	0.405***	0.406***
	(3.478)	(3.388)	(3.490)	(3.486)	(3.496)
*Epu*	-0.003***	-0.003***	-0.003***	-0.003***	-0.003***
	(-7.162)	(-7.162)	(-7.165)	(-7.165)	(-7.166)
*HigInd*	0.074***	0.076***	0.074***	0.074***	0.074***
	(7.772)	(7.956)	(7.754)	(7.749)	(7.762)
*Constant*	-4.279***	-4.287***	-4.280***	-4.279***	-4.281***
	(-20.476)	(-20.463)	(-20.488)	(-20.474)	(-20.498)
*Year*	YES	YES	YES	YES	YES
*Industry*	YES	YES	YES	YES	YES
*Observations*	2,258	2,258	2,258	2,258	2,258
*R2*	0.604	0.604	0.604	0.604	0.604

This table reports the OLS regression results on the relation between VC Quan Zi (*StkQzDum*, *LSQzNum*, *LSInvNum*, *LSInvAmt*, *LSIpoNum*) and stock mispricing (*Mis1*) for the samples with the negative deviation of asset mispricing (Mis1< = 0). All variables are defined in Table 3. Year and industry effects are controlled for in all columns. Robust t statistics are reported in the parentheses below the coefficient estimates, with ***, **, and * denoting significance at 1%, 5%, and 10%, respectively.

### 4.2 Venture Capital Quan Zi’s exit time and asset mispricing

The above empirical results preliminarily verify that VC Quan Zi increases the positive deviation of asset mispricing. VC Quan Zi continuously pushes up stock prices. Logically, it may be to meet the needs of reducing holdings and create favorable exit conditions [40]. As its shareholding decreases, the impact of VC Quan Zi on the positive deviation of asset mispricing will be weakened. Therefore, it is expected that the influence of capital "Quan Zi" on asset mispricing is related to the exit time of VC institutions. Due to the limitation of data availability, we could not accurately obtain the exit time of all VC institutions. Previous literature generally believes that VC institutions still have a significant impact on companies three years after IPO [2, 50]. Feng and Yang [62] found that the lock-in period of most VC institutions is one year, and most VC institutions show a large-scale exit one year after the lock-in period ends. The provisions on restricted stocks can be found in the Company Law of the People’s Republic of China, the department regulations of the China Securities Regulatory Commission (CSRC), the Listing Rules and normative documents of the exchange, as well as the window guidance of the auditor. Generally, the controlling shareholder, the actual controller, and the related party are locked for 36 months from the listing date. Common stockholders (not controlling shareholders, actual controllers, and related parties, and there is no pre-IPO investment) are subject to Article 141 of the Company Law (2013): Shares issued by the company before the share offering shall not be transferred within one year from the date on which the shares of the company are listed on a stock exchange. To implement the requirements of "Several Opinions of The State Council on Promoting the Sustainable and Healthy Development of Venture Capital", the CSRC issued a regulatory question and answer on June 2, 2017, clarifying that it will deal with the limited period of shares of venture capital fund shareholders according to different circumstances: If the issuer has the actual controllers, the venture capital fund shareholders who are not actual controllers shall be locked for one year in accordance with the relevant provisions of Article 141 of the Company Law. For issuers without actual controllers (or it is difficult to judge whether there are actual controlling shareholders), the venture capital fund shareholders who are not actual controllers and within the range of 51% total shareholders holding or more, are no longer required to commit to a 36-month lock-in of their holdings from the listing date. However, this is in accordance with the relevant provisions of Article 141 of the Company law lock for one year.

To examine the dynamic impact of VC Quan Zi on asset mispricing, the following two methods were used for analysis. First, the time interval from the company’s IPO is set as T. It is divided into four periods: 0–12 months, 13–24 months, 25–36 months, and more than 36 months. The first method is to set the four time intervals as dummy variables, 0–12 months as a dummy variable (*Dummy1*), 13–24 months as a dummy variable (*Dummy2*), and 25–36 months as a dummy variable (*Dummy3*). The samples over 36 months are used as the baseline group. The model in Eq (7) was used to investigate.

Column (1) of Table 5 shows that the regression coefficient of the 0–12 month dummy variable (*Dummy1*) is positive at the 1% significance level, the coefficient of the 13–24 month dummy variable (*Dummy2*) is positive at the 5% significance level, and the coefficient of the 25–36 month dummy variable (*Dummy3*) is insignificant. The results indicate that during the lock-in period and the next year, the asset mispricing of the sample firms increased significantly, while asset mispricing decreased with the gradual withdrawal of VC Quan Zi.

**Table 5:** VC Quan Zi’s exit time and asset mispricing.

	Model 7	Model 8		
	(1)	(2)	(3)	(4)
*VARIABLES*	*Mis1*	*Mis1* (0–12 months)	*Mis1* (13–24 months)	*Mis1* (25–36 months)
*Dummy1*	0.056***			
	(4.551)			
*Dummy2*	0.027**			
	(2.364)			
*Dummy3*	0.010			
	(0.951)			
StkQzDum*T		0.022***	0.015***	0.002
		(2.903)	(3.125)	(0.680)
*Control*	YES	YES	YES	YES
*Year*	YES	YES	YES	YES
*Industry*	YES	YES	YES	YES
*Observations*	5,481	1,217	1,692	1,653
*R2*	0.700	0.783	0.732	0.713

This table reports the OLS regression results of dynamic impact of VC Quan Zi on asset mispricing. Year and industry fixed effects are controlled for in all columns. Robust t statistics are reported in the parentheses below the coefficient estimates, with ***, **, and * denoting significance at 1%, 5%, and 10%, respectively.

In the second method, the intersection of the time interval within three years (T) and the VC Quan Zi indicator (*StkQzDum*) are introduced into Eq (8). It is used to investigate the dynamic impact of the exit process of VC Quan Zi on asset mispricing. As shown in Columns (2) to (4) of Table 6, the coefficient of the interaction term (StkQzDum*T) is positive at the 1% significance level in the periods of 0–12 months and 13–24 months, but it is insignificant in the period of 25–36 months. The result indicates that VC Quan Zi promotes the positive deviation of asset mispricing during the period of the potential exit. However, over time, the positive deviation of asset mispricing tends to decrease gradually. This proves the opportunistic motivation of VC Quan Zi to push up the stock price and create favorable conditions for a successful exit.


$${Mis1}_{it}=\beta_{0}+\beta_{1}Dummy1+\beta_{2}Dummy2\text{+}{\beta_{3}Dummy3+\beta_{4}Control}_{it}+{Industry}_{i}{\text{+Year}}_{t}+\varepsilon_{it}$$ (7)

$${Mis1}_{it}=\beta_{0}+\beta_{1}X_{it}+\beta_{2}X_{it}*T\text{+}{\beta_{3}Control}_{it}+{Industry}_{i}{\text{+Year}}_{t}+\varepsilon_{it}$$ (8)

**Table 6:** Differences-in-differences.

	(1)	(2)	(3)
*VARIABLES*	*Mis1*	*Mis1*	*Mis1*
Treat*Post	0.061**	0.055*	0.121***
	(2.238)	(1.721)	(3.220)
*Control*	No	YES	YES
*Year*	No	No	YES
*Firm*	No	No	YES
*Observations*	5,481	5,481	5,481
*R2*	0.031	0.673	0.867

This table reports the DID results on the relation between VC Quan Zi (*StkQzDum*, *LSQzNum*, *LSInvNum*, *LSInvAmt*, *LSIpoNum*) and stock mispricing (*Mis1*) for the samples with the positive deviation of asset mispricing (Mis1> = 0). Robust t statistics are reported in the parentheses below the coefficient estimates, with ***, **, and * denoting significance at 1%, 5%, and 10%, respectively.

### 4.3 Endogeneity correction

Although the above empirical results preliminarily verify and support Hypothesis 1A, it is believed that VC Quan Zi can significantly influence the positive deviation of asset mispricing and aggravate the deviation of the market value of the invested company from its intrinsic value. However, there may be endogeneity problems in VC Quan Zi, which may cause bias in the estimation. To minimize the impact of endogeneity problems, three approaches were adopted in this paper. The first is the difference-in-difference (DID), the second is the propensity score matching analysis (PSM), and the third is the placebo test for randomly shuffling explanatory variables.

#### 4.3.1 Difference-in-difference (DID)

To control the confusing factors of other events in the time series and investigate the influence of VC Quan Zi on the positive deviation of asset mispricing, the difference-in-difference (DID) was further used for verification. VC Quan Zi embodies the important characteristics of Chinese interpersonal relations with a “differential order pattern”. It plays an important role as a supplement to the informal system during the special period of China’s transition economy. “Quan Zi” have different effects on regions with different degrees of marketization. First, the higher the degree of marketization, the stronger the standardized operation of institutions, the more transparent the information, the more frequent the flow of resources, and the capital “Quan Zi” as the complementary effect of informal institutions weakened. However, in regions with a lower degree of marketization, capital is more closely “bonded”, and more information and resources can be obtained through “Quan Zi”. The more obvious the influence of capital “Quan Zi” on the company and the market, the more likely VC Quan Zi is to act as an active “lubricant” [63]. Therefore, we adopt the marketization index of Fan et al. [64] and group the samples according to the median marketization index. Groups with higher marketization than the median are the control group (Treat = 0). Those below the median are the treatment group (Treat = 1). Meanwhile, in 2014, the CSRC issued No.105 order “Interim Measures for the Supervision and Administration of Private Investment Funds”. It officially brought the private capital market into the scope of supervision. Stricter supervision has a direct impact on the four key links (fundraising, investment, management, and withdrawal) of VC institutions. Meanwhile, the development of VC institutions is closely related to regulatory policies. Therefore, we took the implementation of this policy as the impact of exogenous events influenced by VC institutions in recent years and construct the policy shock variable (*Post*, Post = 1 if the time is after 2014, otherwise, it equals 0). Then, we adopted the model in Eq (9) for validation. What we are interested in is the coefficient in front of the interaction term (Treat*Post), which represents the change in the treatment group relative to the less affected control group before the policy shock.


$${M\text{is}1}_{it}=\beta_{0}+\beta_{1}{Treat}_{i}+{\beta_{2}P\text{ost}}_{t}+{\beta_{3}{Treat}_{i}*{P\text{ost}}_{t}+\beta_{4}Control}_{it}+{Firm}_{i}\text{+}Year_{t}+\varepsilon_{it}$$ (9)

The results are reported in Table 6. Columns (1) to (3) represent the results of univariate regression, the regression added control variables, and the regression considered year fixed effects and firm fixed effects, respectively. The results passed the parallel trend test, which is not reported here due to space constraints. The results show that the coefficients of the interaction term (Treat*Post) are all significantly positive. In Column (3), the coefficient of the interaction term (Treat*Post) is 0.121, which is positive at the 1% significance level. This indicates that “Quan Zi” plays a greater role in the treatment group. The positive deviation of asset mispricing of the treatment group significantly increased compared with that of the control group in the same period, further confirming our previous findings.

#### 4.3.2 Propensity score matching analysis (PSM)

We further use propensity score matching analysis (PSM) to reduce the dependence of the estimation on the functional form. First, we adopt the control variables in Eq (6) to perform logit regression. To calculate the propensity score value (PS), the natural logarithm of total assets (*Size*), return on equity (*ROE*), market to book ratio (*BM*), shareholders’ equity concentration (*Hhi*), the logarithm of the number of board members (*BoaNum*), and the dummy variable of whether the company belongs to the high-risk industry (*HigInd*), the logarithm of the number of years from the company’s establishment to IPO (*Age*), China’s Economic Policy Uncertainty Index (Epu), the leverage ratio of the company (*Lev*), the proportion of the company’s independent directors (*IndBNum*), and the shareholding ratio of the controlling shareholder (*First*) are used as matching factors. Then, the repeatable matching method of nearest neighbor matching was adopted and each company invested by “Quan Zi” according to 1:2 was matched to obtain the control group samples. As shown in the results of the mean difference of covariates in the treatment group and the control group, the standard deviation of covariates significantly reduced after matching. The matching was effective. From the graph of the common value range of propensity score, both the control group and the treatment group are in the common value range. The results are not reported here due to space constraints.

Table 7 reports the regression results after matching. As shown in Column (1), the coefficient of the dummy variable (*StkQzDum*) is 0.025, which is significant at the 5% level. This indicates that the degree of asset mispricing significantly increased by 2.5% invested by VC with “Quan Zi” compared to that without “Quan Zi”. Column (2) shows that the degree of asset mispricing increased by 2.4 percentage points when the number of “Quan Zi” increased by 1%.

**Table 7:** Propensity scores matching analysis (PSM).

	(1)	(2)	(3)	(4)	(5)
*VARIABLES*	*Mis1*	*Mis1*	*Mis1*	*Mis1*	*Mis1*
*StkQzDum*	0.025**				
	(2.534)				
*LSQzNum*		0.024***			
		(2.608)			
*LSInvNum*			0.003***		
			(2.628)		
*LSInvAmt*				0.002***	
				(2.665)	
*LSIpoNum*					0.005**
					(2.368)
*Control*	YES	YES	YES	YES	YES
*Year*	YES	YES	YES	YES	YES
*Industry*	YES	YES	YES	YES	YES
*Observations*	3,362	3,362	3,362	3,362	3,362
*R2*	0.740	0.740	0.740	0.740	0.740

This table reports the PSM results on the relation between VC Quan Zi (*StkQzDum*, *LSQzNum*, *LSInvNum*, *LSInvAmt*, *LSIpoNum*) and stock mispricing (*Mis1*) for the samples with the positive deviation of asset mispricing (Mis1> = 0). Robust t statistics are reported in the parentheses below the coefficient estimates, with ***, **, and * denoting significance at 1%, 5%, and 10%, respectively.

As shown in Columns (3) to (5), the power of “Quan Zi” partners also contributes significantly to the degree of asset mispricing. The degree of asset mispricing significantly increased by 0.3 percentage points when the number of “Quan Zi” increased by one unit; the degree of asset mispricing significantly increased by 0.2 percentage points when the investment amount increased by one unit; the degree of asset mispricing significantly increased by 0.5 percentage points when the successful IPO quantity increased by 1%. The results are consistent with the above.

#### 4.3.3 Placebo test

**Fig 1:**
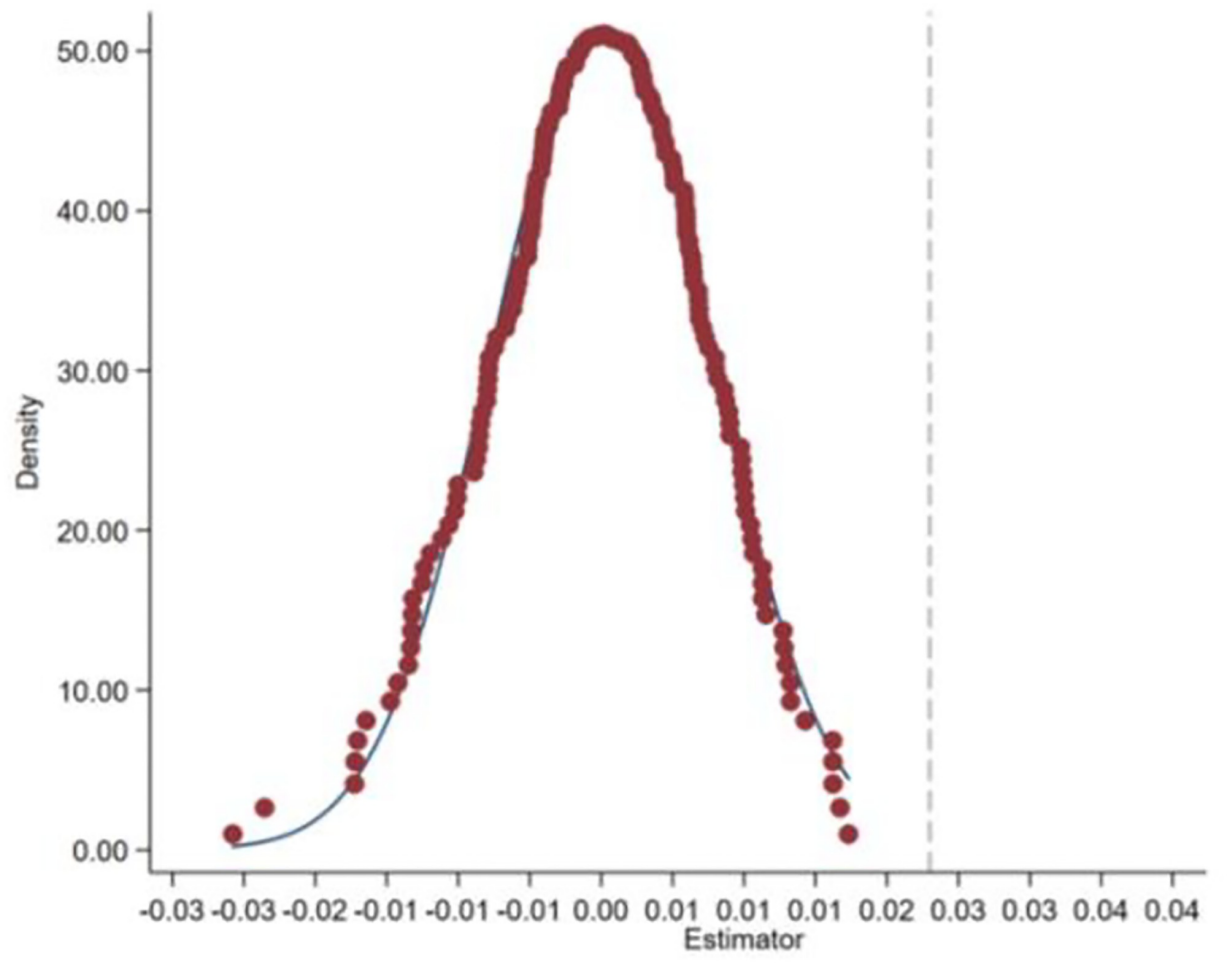
Placebo test of VC Quan Zi and asset mispricing.

To further confirm the causal relationship identification in this paper, following Cao and Zhang [65], we randomly matched the capital "Quan Zi" and the invested company for the placebo test. After randomly matching firms and capital "Quan Zi" 1000 times, the results show that VC Quan Zi has no significant effect on the positive deviation of asset mispricing. Fig 1 shows the regression coefficient distribution of whether the company is invested in by VC Quan Zi (*StkQzDum*) after 1000 random treatments. We found that the coefficient distribution of whether the company is invested in by VC Quan Zi (*StkQzDum*) was concentrated at approximately 0, which is significantly smaller than the estimated value (0.022). Thus, the results of the placebo test provide further evidence that VC Quan Zi pushes up the degree of stock mispricing.

## 5. Mechanism tests

The literature generally classifies the causes of asset mispricing into two categories. The first category includes internal reasons, for example, internal earnings management activities and the quality of accounting information affect asset mispricing in the capital market [14, 66]. The second category is the external reasons, such as investors’ irrational cognition, media power, and the influence of institutional investors [19, 32, 67]. VC institutions are special shareholders that can participate in the operation and management of enterprises [37, 38]. They can control enterprises, and they have information advantages [48, 68]. They may use the relationship network in the market to influence the behavior of market participants [53]. In particular, VC Quan Zi has greater discourse power and social influence than the general VC institutions. Therefore, the influence channels from internal and external aspects were analyzed at the same time.

### 5.1 Venture capital Quan Zi, earnings management and asset mispricing

VC Quan Zi was formed by repeated cooperation. The partners of Quan Zi have long-term relationships with loyalty, trust, and stability. Quan Zi is condensed into a community of interests and has a strong voice. To maximize their interests, they may threaten or collude with the management of companies, conducting earnings manipulation and “window dressing” profits in the process of exiting. Cai [40] found that companies supported by VC had obvious earnings manipulation after listing to meet the needs of share reduction and exit. Based on information asymmetry theory, the quality of accounting information determines the effectiveness of asset pricing. To test whether VC Quan Zi affects the positive deviation of asset mispricing through the channel of earnings management, the real earnings management (REM) index following Roychowdhury [69] was calculated. Additionally, following Li et al. [70], the interaction term was introduced into the model and Eq (10) was used for testing. *X*_*it*_ is the VC Quan Zi indicator, which is characterized by three types of variables, including the dummy variable of whether the company is invested by Quan Zi (*StkQzDum*), the logarithm of the number of Quan Zi (*StkQzNum*), and the power of Quan Zi partners. “Power” is measured by the logarithm of Quan Zi partners’ investment quantity (*StkInvNum*), the logarithm of investment amount (*StkInvAmt*), and the logarithm of IPO quantity (*StkIpoNum*). The coefficient *β*_*1*_ of the interaction term (*REM*_*it*_ * *X*_*it*_) is of interest.


$${Mis1}_{it}=\beta_{0}+{\beta_{1}REM}_{it}{*X}_{it}+{\beta_{2}REM}_{it}+{\beta_{3}X}_{it}+{\beta_{4}Control}_{it}+{Industry}_{i}\text{+}Year_{t}+\varepsilon_{it}$$ (10)

The regression results are reported in Table 8. As shown in Columns (1) to (5), the coefficients of the interaction term are all positive at the 1% significance level, indicating that VC Quan Zi does push up the degree of asset mispricing through the channel of earnings management. In the case of asymmetric market information, the behavior of VC Quan Zi may affect investors’ accurate judgment of the company’s true value, thus raising investors’ expectations to push up stock prices. This may lead to a serious deviation between the market value and the intrinsic value of the invested company.

**Table 8:** VC Quan Zi, earnings management and asset mispricing.

	(1)	(2)	(3)	(4)	(5)
*VARIABLES*	*Mis1*	*Mis1*	*Mis1*	*Mis1*	*Mis1*
StkQzDum* REM	0.232***				
	(5.252)				
LSQzNum * REM		0.139***			
		(2.837)			
LSInvNum * REM			0.031***		
			(5.231)		
LSInvAmt * REM				0.019***	
				(5.107)	
LSIpoNum * REM					0.040***
					(3.597)
*StkQzDum*	0.023**				
	(2.217)				
*LSQzNum*		0.017*			
		(1.877)			
*LSInvNum*			0.003**		
			(2.211)		
*LSInvAmt*				0.002**	
				(2.381)	
*LSIpoNum*					0.002
					(1.172)
*REM*	0.012*	0.082*	0.169***	0.065**	0.179***
	(1.942)	(1.953)	(6.293)	(2.340)	(6.451)
*Control*	YES	YES	YES	YES	YES
*Year*	YES	YES	YES	YES	YES
*Industry*	YES	YES	YES	YES	YES
*Observations*	2,766	2,766	2,766	2,766	2,766
*R2*	0.684	0.683	0.684	0.684	0.683

The table reports the results of the mechanism tests of earnings management. Robust t statistics are reported in the parentheses below the coefficient estimates, with ***, **, and * denoting significance at 1%, 5%, and 10%, respectively.

### 5.2 Venture capital Quan Zi, positive media coverage and asset mispricing

You and Wu [19] found that media sentiment is induced and increases investors’ irrational judgments continuously. In particular, optimistic media sentiment is more likely to lead stock prices to deviate from intrinsic value. Song and Jie [67] pointed out that the larger the media coverage is, the more serious the investors blindly follow the trend, and the higher the degree of asset mispricing. The partners in VC Quan Zi have diverse and wide resource links, which make it easier to mobilize resources. They have strong market power and are more likely to attract media attention. They can even obtain conductive positive reports through interest transmission [71, 72]. Therefore, we use the amount of positive media coverage of the company to measure the company’s positive media coverage. We examine whether it is a channel for VC Quan Zi to improve the degree of positive deviation of asset mispricing. The regression model is Eq (11). *X*_*it*_ is the VC Quan Zi indicator, and *Media*_*it*_ is the amount of positive media coverage of the company. The coefficient *β*_1_ of the interaction term *Media*_*it*_ * *X*_*it*_ is of interest.


$${Mis1}_{it}=\beta_{0}+{\beta_{1}Media}_{it}{*X}_{it}+{\beta_{2}Media}_{it}+{\beta_{3}X}_{it}+{\beta_{4}Control}_{it}+{Industry}_{i}\text{+}Year_{t}+\varepsilon_{it}$$ (11)

Table 9 reports the regression results. As shown in Columns (1) to (5), the coefficients of the interaction term *Media*_*it*_ * *X*_*it*_ are all significantly positive, which indicates that VC Quan Zi does increase the amount of positive media coverage. It gives optimistic signals to market investors, thus causing stock price overvaluation. The development of China’s capital market started late; therefore, the channels to obtain information are limited, investors are still inexperienced, and they are easily influenced by dominant opinions. High media attention and strong positive signals easily attract the attention of investors [73], causing a herd effect and pushing up the stock price bubble.

**Table 9:** VC Quan Zi, positive media coverage and asset mispricing.

	(1)	(2)	(3)	(4)	(5)
*VARIABLES*	*Mis1*	*Mis1*	*Mis1*	*Mis1*	*Mis1*
StkQzDum* Media	0.019*				
	(1.766)				
LSQzNum *Media		0.022***			
		(2.877)			
LSInvNum* Media			0.003**		
			(2.445)		
LSInvAmt * Media				0.002**	
				(2.451)	
LSIpoNum* Media					0.005**
					(2.449)
*StkQzDum*	0.017*				
	(1.840)				
*LSQzNum*		0.022***			
		(2.715)			
*LSInvNum*			0.003**		
			(2.509)		
*LSInvAmt*				0.002**	
				(2.480)	
*LSIpoNum*					0.003
					(1.638)
*Media*	0.038***	0.049***	0.050***	0.039***	0.051***
	(3.658)	(6.830)	(7.109)	(4.569)	(7.114)
*Control*	YES	YES	YES	YES	YES
*Year*	YES	YES	YES	YES	YES
*Industry*	YES	YES	YES	YES	YES
*Observations*	4,289	4,289	4,289	4,289	4,289
*R2*	0.641	0.642	0.641	0.641	0.641

The table reports the results of the mechanism tests of positive media coverage. Robust t statistics are reported in the parentheses below the coefficient estimates, with ***, **, and * denoting significance at 1%, 5%, and 10%, respectively.

### 5.3 Venture capital Quan Zi, institutional investors, and asset mispricing

There are different views on the role that institutional investors play in asset mispricing. One view supports that institutional investors are rational investors and arbitrageurs, which are conducive to the return of stock prices to their true value. Companies with more shareholding of institutional investors have higher pricing efficiency [74]. The other view is that institutional investors do not always oppose asset mispricing, but rather they take advantage of such mispricing and ride the stock market bubble for their benefit. For example, Gompers and Metrick [75] found that the shareholding of institutional investors was positively correlated with stock returns. VC Quan Zi has strong market power and network connections with highly qualified market participants and regulators, such as the CSRC, underwriters, law teams, accounting firms, and analysts. Additionally, VC Quan Zi is undoubtedly favored by more institutional investors. Next, whether VC Quan Zi contributes to the positive deviation of asset prices through institutional investors was examined. The regression model is Eq (12). *X*_*it*_ is the proxy of VC Quan Zi, and *FDs*_*it*_ is the shareholding of institutional investors. The coefficients *β*_1_ of the interaction term *Fds*_*it*_ * *X*_*it*_ are of interest.


$${M\text{is}1}_{it}=\beta_{0}+{\beta_{1}Fds}_{it}{*X}_{it}+{\beta_{2}Fds}_{it}+{\beta_{3}X}_{it}+{\beta_{4}Control}_{it}+{Industry}_{i}\text{+}Year_{t}+\varepsilon_{it}$$ (12)

Table 10 reports the regression results. As shown in Columns (1) to (5), the coefficients of the interaction term (*Fds*_*it*_ * *X*_*it*_) are all positive at the 1% significance level, indicating that VC Quan Zi does cause the overvaluation of stock prices through the influence channel of institutional investors. On the one hand, VC Quan Zi attracts institutional investors to increase their holdings. It shows a certain positive feedback effect, which causes the irrational following of investors in the market and promotes the emergence and expansion of the stock market bubble. On the other hand, the team of institutional managers in China’s capital market is relatively young [76]. They are more willing to increase their holdings of overpriced stocks and take advantage of such mispricing to ride the stock market bubble for their own benefit, further boosting the positive deviation of asset prices.

**Table 10:** VC Quan Zi, institutional investors, and asset mispricing.

	(1)	(2)	(3)	(4)	(5)
*VARIABLES*	*Mis1*	*Mis1*	*Mis1*	*Mis1*	*Mis1*
StkQzDum* Fds	0.003***				
	(5.587)				
LSQzNum * Fds		0.003***			
		(5.024)			
LSInvNum * Fds			0.001***		
			(6.417)		
LSInvAmt * Fds				0.001***	
				(6.649)	
LSIpoNum * Fds					0.001***
					(6.728)
*StkQzDum*	0.008*				
	(1.954)				
*LSQzNum*		0.019***			
		(2.817)			
*LSInvNum*			0.002**		
			(2.416)		
*LSInvAmt*				0.001**	
				(2.491)	
*LSIpoNum*					0.003**
					(2.136)
*Fds*	0.002***	0.005***	0.005***	0.003***	0.005***
	(4.477)	(11.641)	(11.587)	(6.753)	(11.700)
*Control*	YES	YES	YES	YES	YES
*Year*	YES	YES	YES	YES	YES
*Industry*	YES	YES	YES	YES	YES
*Observations*	4177	4177	4177	4177	4177
*R2*	0.779	0.779	0.779	0.779	0.779

The table reports the results of the mechanism tests of institutional investors. Robust t statistics are reported in the parentheses below the coefficient estimates, with ***, **, and * denoting significance at 1%, 5%, and 10%, respectively.

### 5.4 Venture Capital Quan Zi, equity liquidity and asset mispricing

The liquidity of stocks reflects the characteristics of stock realization. The stronger the liquidity, the higher the attention of investors, and the more likely the stock to be pushed up. Following Amihud [77], we use the illiquidity index *Amihud* to verify whether VC Quan Zi causes stock price overvaluation through the channel of stock liquidity improvement. The illiquidity index *Amihud* has been widely used by Chinese scholars in academia on stock liquidity [78, 79]. It measures the liquidity of stocks by the ratio of the absolute stock return to the transaction amount. The smaller the index is, the higher the liquidity. The regression model is Eq (13). *X*_*it*_ is the VC Quan Zi indicator, and *Amihud*_*t*_ is the illiquidity indicator calculated by quarter data. The coefficient *β*_1_ of the interaction term (*Amihud*_*t*_ * *X*_*it*_) is of interest. It is expected to be significantly negative.


$${M\text{is}1}_{it}=\beta_{0}+{\beta_{1}{Amihud}_{t}}_{}{*X}_{it}+{\beta_{2}{Amihud}_{t}}_{}+{\beta_{3}X}_{it}+{\beta_{4}Control}_{it}+{Industry}_{i}\text{+}Year_{t}+\varepsilon_{it}$$ (13)

Table 11 reports the regression results. As shown in Columns (1) to (5), the coefficients of the illiquidity index (*Amihud*_*t*_) are negative at the 1% significance level, indicating that the increase in stock liquidity causes the overvaluation of stock prices. At the same time, the coefficients of the interaction term (*Amihud*_*t*_ * *X*_*it*_) of the VC Quan Zi indicator and the illiquidity indicator are both significantly negative, which is consistent with our expectations and the above analysis. VC Quan Zi condenses into a community of interests. It uses diversified resource links and powerful market forces to attract highly qualified institutional investors and highly influential media attention and market participation. In addition, it uses information asymmetry in the market to boost the expansion of asset bubbles and improve the degree of positive deviation of asset mispricing through the improvement of stock liquidity.

**Table 11:** VC Quan Zi, equity liquidity and asset mispricing.

	(1)	(2)	(3)	(4)	(5)
*VARIABLES*	*Mis1*	*Mis1*	*Mis1*	*Mis1*	*Mis1*
StkQzDum* Amihud	-0.342***				
	(-2.645)				
LSQzNum * Amihud		-0.398*			
		(-1.958)			
LSInvNum * Amihud			-0.151**		
			(-2.476)		
LSInvAmt * Amihud				-0.134***	
				(-2.604)	
LSIpoNum * Amihud					-0.158**
					(-1.979)
*StkQzDum*	0.029				
	(1.169)				
*LSQzNum*		0.023			
		(0.681)			
*LSInvNum*			0.011		
			(1.001)		
*LSInvAmt*				0.011	
				(1.228)	
*LSIpoNum*					-0.001
					(-0.047)
*Amihud*	-0.332***	-0.359***	-0.338***	-0.331***	-0.365***
	(-4.521)	(-4.751)	(-4.554)	(-4.512)	(-4.741)
*Control*	YES	YES	YES	YES	YES
*Year*	YES	YES	YES	YES	YES
*Industry*	YES	YES	YES	YES	YES
*Observations*	3789	3789	3789	3789	3789
*R2*	0.344	0.343	0.344	0.344	0.343

The table reports the results of the mechanism tests of equity liquidity. Robust t statistics are reported in the parentheses below the coefficient estimates, with ***, **, and * denoting significance at 1%, 5%, and 10%, respectively.

## 6. Robustness test

Three methods for the robustness test were used, including replacing the calculation method of asset mispricing, measuring from the perspective of excess abnormal returns, and adding other control variables.

### 6.1 Replacing the calculation method of asset mispricing

Because the intrinsic value of a company is difficult to directly observe, the calculation method of asset mispricing has a certain complexity and diversity. To ensure the robustness of our results, we followed Rhodes-Kropf et al. [58] and used the decomposition method of the market-to-book ratio to calculate the degree of asset mispricing. Tables 12 and 13 report the estimation results after replacing the calculated indicators. As shown in Columns (1)—(5) of Table 12, the coefficients of VC Quan Zi are positive at the 1% significance level. In Columns (1)—(5) of Table 13, the coefficients of VC Quan Zi are insignificant. This shows that after replacing the calculation method of asset mispricing, the result is still consistent with the previous finding.

**Table 12:** VC Quan Zi and asset mispricing (positive)—Alternative indicators.

	(1)	(2)	(3)	(4)	(5)
*VARIABLES*	*Mis2*	*Mis2*	*Mis2*	*Mis2*	*Mis2*
*StkQzDum*	0.019***				
	(2.746)				
*LSQzNum*		0.013***			
		(5.827)			
*LSInvNum*			0.003***		
			(3.839)		
*LSInvAmt*				0.002***	
				(3.851)	
*LSIpoNum*					0.004***
					(3.101)
*Control*	YES	YES	YES	YES	YES
*Year*	YES	YES	YES	YES	YES
*Industry*	YES	YES	YES	YES	YES
*Observations*	5,481	5,481	5,481	5,481	5,481
*R2*	0.788	0.790	0.788	0.788	0.788

This table reports the OLS regression results of a different calculation method of asset mispricing for the samples with the positive deviation of asset mispricing (Mis1> = 0). Year and industry fixed effects are controlled for in all columns. Robust t statistics are reported in the parentheses below the coefficient estimates, with ***, **, and * denoting significance at 1%, 5%, and 10%, respectively.

**Table 13:** VC Quan Zi and asset mispricing (negative)—Alternative indicators.

	(1)	(2)	(3)	(4)	(5)
*VARIABLES*	*Mis2*	*Mis2*	*Mis2*	*Mis2*	*Mis2*
*StkQzDum*	0.002				
	(0.292)				
*LSQzNum*		-0.006			
		(-1.295)			
*LSInvNum*			0.001		
			(0.422)		
*LSInvAmt*				0.001	
				(0.383)	
*LSIpoNum*					0.001
					(0.539)
*Control*	YES	YES	YES	YES	YES
*Year*	YES	YES	YES	YES	YES
*Industry*	YES	YES	YES	YES	YES
*Observations*	2,258	2,258	2,258	2,258	2,258
*R2*	0.819	0.819	0.819	0.819	0.819

This table reports the OLS regression results of a different calculation method of asset mispricing for the samples with the negative deviation of asset mispricing (Mis1< = 0). Year and industry fixed effects are controlled for in all columns. Robust t statistics are reported in the parentheses below the coefficient estimates, with ***, **, and * denoting significance at 1%, 5%, and 10%, respectively.

### 6.2 Measure from the angle of excess abnormal returns

Another common method to measure asset mispricing is to combine the principle of asset pricing and investigate it from the perspective of market anomaly. In this part, we followed the relevant research [2, 20, 21] and measure the excess abnormal return of the company’s stock price. We adopted CAPM (1964), Fama-Frech three-factor model (1993) and Carhart four-factor model (1997) to verify and use the monthly stock return rate to construct the calendar-time portfolio, comparing the excess return α. We selected monthly observations from January 2009 to December 2019 and divided the IPO sample companies into three groups: no Quan Zi, large Quan Zi and small Quan Zi. Large and small Quan Zi groups are divided according to the median number of "Quan Zi" of listed companies. Those below the median are defined as small Quan Zi, and those above the median are large Quan Zi. Based on the monthly return rate, the stock portfolio listed within 18 months was constructed, and three hedge portfolios were established. That is, buy the small Quan Zi and sell the no Quan Zi portfolio, buy the large Quan Zi and sell the no Quan Zi portfolio, buy the large Quan Zi and sell the small Quan Zi portfolio. Then, we calculated the return of the rate of the portfolios. Circulation market value is the product of the portfolio’s closing price at the end of the last month and the number of shares outstanding. We also constructed the stock portfolio listed at 24 months and 36 months, calculating its return rate based on the monthly return rate for verification. The results are reported in Table 14, which is consistent with the previous finding.

**Table 14:** Robustness test of CAPM, three-factor and four-factor models.

Hedging portfolio	Small Quan Zi- no Quan Zi	Large Quan Zi- no Quan Zi	Large Quan Zi- small Quan Zi
Panel A: Market model: Equal-weighted portfolios			
*α*	0.007**	0.010**	0.003
	(2.301)	(2.430)	(0.920)
*Mrk-Rf*	-0.021	-0.030	-0.010
	(-0.445)	(-0.473)	(-0.182)
Panel B: Market model: Value-weighted portfolios			
*α*	0.014***	0.017**	0.003
	(2.635)	(2.181)	(0.430)
*Mrk-Rf*	0.063	0.110	0.048
	(0.785)	(0.943)	(0.456)
Panel C: Three-factor model: Equal-weighted portfolios			
*α*	0.009***	0.012***	0.003
	(2.820)	(2.746)	(0.853)
*Mrk-Rf*	-0.012	-0.043	-0.031
	(-0.249)	(-0.651)	(-0.563)
*SMB*	-0.243**	-0.184	0.060
	(-2.486)	(-1.352)	(0.525)
*HML*	-0.260***	-0.318**	-0.058
	(-2.847)	(-2.511)	(-0.548)
Panel D: Three-factor model: Value-weighted portfolios			
*α*	0.017***	0.021***	0.004
	(3.330)	(2.818)	(0.608)
*Mrk-Rf*	0.083	0.168	0.085
	(1.038)	(1.419)	(0.766)
*SMB*	-0.503***	-0.765***	-0.262
	(-3.089)	(-3.171)	(-1.159)
*HML*	-0.526***	-0.634***	-0.108
	(-3.465)	(-2.820)	(-0.513)
Panel E: Four-factor model: Equal-weighted portfolios			
*α*	0.009***	0.012***	0.003
	(2.802)	(2.741)	(0.837)
*Mrk-Rf*	-0.007	-0.017	-0.009
	(-0.148)	(-0.245)	(-0.164)
*SMB*	-0.243**	-0.179	0.064
	(-2.464)	(-1.321)	(0.565)
*HML*	-0.253***	-0.277**	-0.024
	(-2.689)	(-2.147)	(-0.225)
*MOM*	0.034	0.199	0.165
	(0.336)	(1.430)	(1.414)
Panel F: Four-factor model: Value-weighted portfolios			
*α*	0.017***	0.021***	0.004
	(3.359)	(2.807)	(0.590)
*Mrk-Rf*	0.054	0.206*	0.152
	(0.659)	(1.685)	(1.349)
*SMB*	-0.508***	-0.757***	-0.249
	(-3.132)	(-3.146)	(-1.124)
*HML*	-0.569***	-0.574**	-0.005
	(-3.673)	(-2.497)	(-0.023)
*MOM*	-0.213	0.291	0.504**
	(-1.278)	(1.173)	(2.212)

This table reports the results of Market model, Three-factor model and Four-factor model from the angle of excess abnormal returns. Robust t statistics are reported in the parentheses below the coefficient estimates, with ***, **, and * denoting significance at 1%, 5%, and 10%, respectively.

### 6.3 Add other control variables

The degree of asset mispricing may be influenced by market sentiment [15, 80]. Due to the complexity of measuring investor sentiment, each proxy variable itself may have a certain leading-lag effect. Following Baker and Wurgler [81], we selected the current and lag values of five indicators, including the discount of closed-end funds, monthly IPO quantity, IPO return of rate, monthly new account number of A-shares, and turnover rate. After eliminating the influence of the macroeconomy, the quarterly investor sentiment index (*Mood*) calculated by principal component analysis (PCA) was introduced as a new control variable into the basic regression. Following You and Wu [19], we also selected the stock closing price at the end of the quarter (*CloPri*) as a new control variable. It was used to control the possible influence of price momentum or reversal effect on asset mispricing. Following Chen et al. [82], we calculated the negative skew index of the quarterly rate of return on trading days (*Ncskew*) and used it as a proxy for the rapid rise and fall of stock prices. It was added to the regression as a control variable to control the impact of events occurring during the study. The regression results are reported in Tables 15 and 16. The results are consistent with the findings of the basic regression after further controlling the above variables.

**Table 15:** VC Quan Zi and asset mispricing (positive)—Adding other control variables.

	(1)	(2)	(3)	(4)	(5)
*VARIABLES*	*Mis1*	*Mis1*	*Mis1*	*Mis1*	*Mis1*
*StkQzDum*	0.025***				
	(3.063)				
*LSQzNum*		0.009***			
		(3.541)			
*LSInvNum*			0.003***		
			(3.658)		
*LSInvAmt*				0.002***	
				(3.731)	
*LSIpoNum*					0.004**
					(2.506)
*Control*	YES	YES	YES	YES	YES
*Year*	YES	YES	YES	YES	YES
*Industry*	YES	YES	YES	YES	YES
*Observations*	4,565	4,565	4,565	4,565	4,565
*R2*	0.705	0.706	0.706	0.706	0.705

This table reports the OLS regression results of adding other control variables for the samples with the positive deviation of asset mispricing (Mis1> = 0). Year and industry fixed effects are controlled for in all columns. Robust t statistics are reported in the parentheses below the coefficient estimates, with ***, **, and * denoting significance at 1%, 5%, and 10%, respectively.

**Table 16:** VC Quan Zi and asset mispricing (negative)—Adding other control variables.

	(1)	(2)	(3)	(4)	(5)
*VARIABLES*	*Mis1*	*Mis1*	*Mis1*	*Mis1*	*Mis1*
*StkQzDum*	0.002				
	(0.382)				
*LSQzNum*		-0.010			
		(-1.627)			
*LSInvNum*			0.001		
			(0.403)		
*LSInvAmt*				0.001	
				(0.480)	
*LSIpoNum*					0.001
					(0.271)
*Control*	YES	YES	YES	YES	YES
*Year*	YES	YES	YES	YES	YES
*Industry*	YES	YES	YES	YES	YES
*Observations*	1,914	1,914	1,914	1,914	1,914
*R2*	0.633	0.634	0.633	0.633	0.633

This table reports the OLS regression results of adding other control variables for the samples with the negative deviation of asset mispricing (Mis1< = 0). Year and industry fixed effects are controlled for in all columns. Robust t statistics are reported in the parentheses below the coefficient estimates, with ***, **, and * denoting significance at 1%, 5%, and 10%, respectively.

## 7. Conclusion and policy implications

The essence of capital is to pursue profit. VC needs to reduce its holdings in the secondary market to obtain high profits. Stock price is naturally the focus of its attention. What is the logic of the role of VC Quan Zi in the capital market in order to create favorable conditions for a smooth exit? Will there be an unfair problem of capital misallocation in order to pursue profits?

Using the syndicate investment data of China’s venture capital institutions from 2009 to 2019, we constructed the proxy of VC Quan Zi and further identified the VC Quan Zi-backed firms in the year of listing and the first two years. Given the background of the Chinese "Quan Zi" culture with a "differential order pattern", this paper investigates how Venture Capital Quan Zi affects the stock mispricing of invested companies. We documented that Venture Capital Quan Zi significantly increases the positive deviations of stock prices but has no obvious influence on the negative deviations, showing an asymmetric effect on stock mispricing. In addition, the impact of VC Quan Zi on the positive deviation is dynamic. During the lock-up period and the second year of the lock-up period, asset mispricing was aggravated, and the positive deviation degree gradually reduced with the withdrawal of its holdings. This reveals the opportunistic motivation of VC Quan Zi. To maximize their own interests and realize the successful exit, VC Quan Zi promotes the stock price bubble, exacerbates the expansion of the market value of enterprises and damages the fairness of the market. The "capital Quan Zi" influences the allocation of funds to VC Quan Zi- backed firms, which obviously goes against the fundamental logic of market development.

Further mechanism tests suggest that VC Quan Zi affects stock mispricing from both internal and external channels. On the one hand, Venture Capital Quan Zi increases a company’s earnings manipulation, thus raising investors’ expectations to push up stock prices. On the other hand, Venture Capital Quan Zi boosts the stock price through market reaction channels, increasing institutional investors’ shareholdings, positive media coverage and stock liquidity.

China’s capital market has a short development history and is in the transition period from a planned economy to a market economy. The traditional culture of Chinese society has a profound impact on the market. Our research not only contributes to the thinking of efficiency and fairness, but also has important implications for investor protection and capital market supervision.

The "Quan Zi culture" of a "differential order pattern" is rooted in every aspect of Chinese society. As a special informal institutional arrangement, "Quan Zi" plays an important role in the capital market. We found that VC Quan Zi drives the market value of enterprises to deviate seriously from the fundamental value through earnings management, positive media coverage, improving stock liquidity and other perspectives, and this effect is more obvious in the exit stage of interest. It can be seen that the power of capital, directly or indirectly, affects market prices in a secretive way, which is obviously unfair. For the developing Chinese capital market, the overvaluation of stock prices related to the capital "Quan Zi" is very likely to attract the attention of investors and change the reasonable expectations of the market, especially by pushing up stock prices in an abnormal way. Stock prices are prone to rise and fall. In the long run, it is not only detrimental to the appreciation of the market value of enterprises, but also aggravates the loss of investors and affects the stability of the market. The phenomenon of cronies and cliques in VC Quan Zi may lead to the undesirable social atmosphere of the privatization of power, and the strong market forces may also boost the capital market bubble. It is suggested that the regulatory authorities should strengthen the supervision of VC institutions from the perspective of capital "Quan Zi", standardize the market boundary of capital power, guide the rational allocation of funds, and protect the interests of investors.

## Supporting information

S1 Data (RAR)
